# High incidence of azole resistance among *Candida albicans* and *C. glabrata* isolates in Northeastern Iran 

**DOI:** 10.18502/cmm.7.3.7801

**Published:** 2021-09

**Authors:** Hossein Zarrinfar, Zahra Kord, Abdolmajid Fata

**Affiliations:** 1 Allergy Research Center, Mashhad University of Medical Sciences, Mashhad, Iran; 2 Department of Parasitology and Mycology, Faculty of Medicine, Mashhad University of Medical Sciences, Mashhad, Iran; 3 Cutaneous Leishmaniasis Research Center, Mashhad University of Medical Sciences, Mashhad, Iran

**Keywords:** Azole, Northeastern Iran, Resistance, Sensitivity, Vulvovaginal candidiasis

## Abstract

**Background and Purpose:**

Resistance to antifungal drugs is increasing among *Candida* isolates from patients with vulvovaginal candidiasis (VVC). Lack of correct diagnosis of *Candida* causing VVC and the experimental use of antifungal drugs are the main causes of this resistance. This study aimed to determine the susceptibility of antifungal drugs against *Candida* species isolated from VVC in Northeastern Iran.

**Materials and Methods:**

Among women suspected of VVC, 189 vaginal discharge specimens were evaluated. *Candida* isolates detected by polymerase chain reaction-restriction fragment length polymorphism were examined by standard antifungal disk diffusion susceptibility testing method for voriconazole, itraconazole, fluconazole, and ketoconazole. The susceptibility pattern of these antifungals was reported as sensitive, susceptible dose-dependent, and resistant. The results were evaluated by SPSS software and analyzed by Pearson chi-squared test.

**Results:**

Among the vaginal specimens, 108 out of 189 *Candida* isolates were identified as *C. albicans*, *C. glabrata*, *C. kefyr*, *C. parapsilosis*, and *C. tropicalis*. The susceptibility rates of *Candida* isolates to voriconazole, ketoconazole, itraconazole, and fluconazole were 92.6%, 90.7%, 68.5%, and 63.9%, respectively. Moreover, the resistance rates to fluconazole, itraconazole, ketoconazole, and itraconazole were 15.7%, 8.3%, 1.9%, and 1.9%, respectively. The *C. glabrata* and *C. albicans* isolates were resistant to antifungal discs among 93% and 20% of the specimens, respectively.

**Conclusion:**

The *C. glabrata* and *C. albicans* species showed the highest resistance to antifungal drugs. Furthermore, *Candida* isolates showed the highest sensitivity to voriconazole and ketoconazole and the lowest sensitivity to fluconazole.

## Introduction

Vulvovaginal candidiasis (VVC) is one of the most common clinical forms of candidiasis among 10% of women during childbearing age that can be developed in a recurrent form [ 1 , 2 ]. The VVC is characterized by multiple symptoms, such as itching, dyspareunia, pruritus, and erythema, which may decrease the life quality of women. Diabetes mellitus, overuse of antibiotics, and pregnancy can increase the complications and severity of this infection [ 3 ].

In most studies, *Candida albicans* has been reported as the most common cause of this infection, compared to other *Candida* species [ 4 , 5 ]. However, the prevalence of non-albicans *Candida* (NAC) spp. including *C. glabrata*, *C. krusei*, and *C. parapsilosis* is increasing [ 6 ]. The reason for the increase in non-albicans spp. is the lack of accurate diagnosis of the causes of vaginitis, and improper use of antifungal medicines [ 2 , 6 ]. Due to the emerging resistance of *Candida* isolates to the limited antifungals available in the clinical setting, it is essential to establish in vitro antifungal susceptibility testing before the treatment of *Candida* infections, particularly VVC. Therefore, accurate identification of the causative agents of this infection and determination of antifungal susceptibility testing for *Candida* isolates can be effective in reducing the drug resistance [ 7 ]. 

Among the antifungal agents with the highest resistance, azoles resistance was the most prevalent [ 8 ]. Although antifungal drug resistance has been frequently reported among NAC spp., the reports of VVC infection caused by fluconazole-resistant *C. albicans* have been rare [ 6 , 9 , 10 ]. There were only a few Iranian studies available, especially in Northeastern Iran on susceptibility testing for *Candida* isolates collected from VVC. Hence, the present study aimed to perform antifungal susceptibility tests on *Candida* isolates obtained from women with VVC by disk diffusion technique.

## Materials and Methods

This study was approved by the Ethics Committee (ethics code: IR.MUMS.fm.REC.1394.204).

In this study, 108 *Candida* isolates were obtained from 189 vaginal discharge specimens of women suspected of VVC (determined by a midwife or gynecologist) and referred to the health centers of Mashhad University of Medical Sciences, Mashhad, Iran to be examined. According to the above, the symptoms were pruritus, vaginal soreness, dyspareunia, external dysuria, and abnormal vaginal discharge. As described previously, the swabs were examined for direct microscopy using potassium hydroxide, cultivated on sabouraud dextrose agar, and the *Candida* colonies were purified using CHROMagar *Candida* [ 11 ].

At the first stage of this study, *Candida* isolates were identified using the polymerase chain reaction-restriction fragment length polymorphism method using Msp I enzyme [ 11 ]. Based on the inclusion criteria, only the purified and identified *Candida* isolates obtained from women affected with VVC were included in the study. The exclusion criteria were the contamination of *Candida* isolates with saprobe fungi.

According to the results, 84, 15, 6, 2, and 1 of the isolated spp. of *Candida* were *C. albicans*, *C. glabrata*, *C. kefyr*, *C. parapsilosis*, and *C. tropicalis*, respectively. To perform antifungal susceptibility testing, the disk diffusion method was performed according to the M44-A2 protocol [ 12 ]. Briefly, Müller Hinton agar (Sigma, Germany) base medium was prepared according to the instructions of the manufacturer and distributed on plates. Yeast suspensions prepared with standard McFarland 0.5 turbidity were cultured uniformly using sterile swabs on plates containing Mueller-Hinton agar (with 2% glucose and 0.5 μg/ml methylene blue).

After 15 min, when the culture medium was dried, commercial disks containing voriconazole, itraconazole, fluconazole, and ketoconazole (Liofilchem, Italy) were placed on the plate. The plates were incubated for 24 h at 35 °C, and the diameter of the growth inhibition zone around each disk was measured. Fluconazole-, itraconazole-, ketoconazole-, and voriconazole-impregnated paper disks contained 100, 50, 15, and 1 μg of antifungals, respectively. The zone diameters were measured. According to the table of antifungal discs recommended by the instructions of the manufacturer (Liofilchem, Italy), their antifungal test profile was reported as sensitive, susceptible dose-dependent, and resistant. The results were analyzed in SPSS software (version 16) using Pearson’s chi-squared test.

## Results

The results of antifungal susceptibility testing of four antifungal agents, fluconazole, ketoconazole, itraconazole, and voriconazole, are summarized in Table 1. 

**Table 1:** The results of antifungal susceptibility testing in *Candida* isolates obtained from vulvovaginal candidiasis (VVC) for 4 antifungals of fluconazole, ketoconazole, itraconazole and voriconazole

Susceptibility profile	Antifungal agents			
	Fluconazole	Ketoconazole	Itraconazole	Voriconazole
Sensitive (%)	63.9	90.7	68.6	92.7
Susceptible dose-dependent (%)	20.4	7.4	23.1	5.5
Resistant (%)	15.7	1.9	8.3	1.8

According to this Table, *Candida* isolates showed the highest sensitivity to voriconazole and ketoconazole. Furthermore, they showed the highest drug resistance to fluconazole and itraconazole, in that order. The results of the antifungal profile of different *Candida* spp. are shown separately in Table 2. Based on these results, the highest rates of sensitivity and resistance of *C. albicans* and *C. glabrata* to fluconazole were 58.3% and 9.3%, respectively.

**Table 2:** The results of antifungal susceptibility test of fluconazole, ketoconazole, itraconazole and voriconazole on different *Candida* species isolated from VVC

*Candida* spp. (No.)	Antifungal agents											
	Fluconazole			Ketoconazole			Itraconazole			Voriconazole		
	S (%)	SDD (%)	R (%)	S (%)	SDD (%)	R (%)	S (%)	SDD (%)	R (%)	S (%)	SDD (%)	R (%)
*C. albicans (84)*	63 (58.3)	14 (12.9)	7 (6.5)	77 (71.3)	5 (4.7)	2 (1.8)	61 (56.5)	18 (16.7)	5 (4.8)	78 (72.3)	4 (3.7)	2 (1.8)
*C. glabrata (15)*	3 (2.8)	2 (1.8)	10 (9.3)	13 (12.1)	2 (1.8)	0 (0)	4 (3.7)	7 (6.4)	4 (3.6)	15 (14)	0 (0)	0 (0)
*C. kefyr (6)*	2 (1.9)	4 (3.8)	0 (0)	5 (4.6)	1 (0.9)	0 (0)	6 (5.6)	0 (0)	0 (0)	4 (3.7)	2 (1.8)	0 (0)
*C. parapsilosis (6)*	0 (0)	2 (1.8)	0 (0)	2 (1.8)	0 (0)	0 (0)	2 (1.8)	0 (0)	0 (0)	2 (1.8)	0 (0)	0 (0)
*C. tropicalis (1)*	1 (0.9)	0 (0)	0 (0)	1 (0.9)	0 (0)	0 (0)	1 (0.9)	0 (0)	0 (0)	1 (0.9)	0 (0)	0 (0)
Total	69 (63.9)	22 (20.4)	17 (15.7)	98 (90.7)	8 (7.4)	2 (1.9)	74 (68.6)	25 (23.1)	9 (8.3)	100 (92.7)	6 (5.5)	2 (1.8)

S, sensitive; SDD, susceptible dose-dependent; R, resistant

The highest rates of sensitivity and resistance of *C. albicans* to ketoconazole were 71.3% and 1.8%, respectively. Moreover, the highest rates of sensitivity and resistance of *C. albicans* to itraconazole were 56.5% and 4.8%, respectively. In addition, the highest rates of sensitivity and resistance of *C. albicans* to voriconazole were 72.3% and 1.8%, respectively. However, with the exception of fluconazole (*P*=0.001), there was no significant difference between various spp. of *Candida* and the antifungals of clotrimazole (*P*=0.994), itraconazole (*P*=0.146), and voriconazole (*P*=0.596).

## Discussion

Due to the limited access to some antifungal agents in many parts of the world, the azole antifungals are the most frequent class used to treat *Candida* infections. Therefore, such antifungals are often preferred for the treatment of various *Candida* infections as they are available, inexpensive, exhibit limited toxicity, and are administrated orally [ 9 ]. On the other hand, there are an incredibly large number of documents about the intrinsic and acquired resistance to azole antifungals among some *Candida* spp. [ 5 ]. However, it is essential to minimize such resistance emergence to both preserve and improve the azole class of antifungals for the treatment of *Candida* infections.

One way to reduce antifungal resistance is to establish in vitro antifungal susceptibility testing prior to antifungal therapy. Therefore, the present study aimed to evaluate the antifungal susceptibility of *Candida* isolates obtained from women with VVC in Mashhad, Northeastern Iran. Although according to the extensive literature, *C. albicans* is the most common causative agent of VVC, *C. glabrata* is reported as the most common NAC spp. [ 4 , 11 ]. However, drug resistance has often been reported in NAC spp., compared to *C. albicans* [ 9 ].

In the present study, although *C. albicans* (78%) was the predominant causative agent of VVC, *C. glabrata* (14%) was the most common NAC spp. In other parts of this region, the spp. distribution has a similar pattern [ 4 , 11 , 13 ]. Therefore, most patterns of antifungal results have been reported for these dominant spp. In the present study, 92% and 90% of *Candida* isolates were sensitive to voriconazole and ketoconazole, respectively.

Among the studied spp., *C. albicans* and *C. glabrata* had the highest rates of sensitivity to voriconazole that were 72.3% and 14%, respectively. Furthermore, the same spp. showed similar sensitivity rates to ketoconazole that were 71.3% and 12.1%, respectively. However, the resistance rate of *C. albicans* isolates against ketoconazole and voriconazole was 1.8%.

In a study conducted by Khan et al. in Lebanon, voriconazole was more effective (97.5%) against *C. albicans* spp., compared to fluconazole (90%) and itraconazole (87.5%) [ 14 ]. In a case reported by Adampour et al., ketoconazole was more effective against *Pichia* fermentans (*C. firmentaria*, a variety of firmentaria) isolated from a vaginitis, compared to fluconazole, itraconazole, and voriconazole. Therefore, it is better to examine and evaluate every *Candida* isolates via an in vitro antifungal susceptibility test before treatment [ 10 ].

In the current study, *Candida* isolates also showed the highest rates of antifungal resistance to fluconazole (15.7%) and itraconazole (8.3%). Moreover, *C. glabrata* and *C. albicans* had the highest resistance rates against fluconazole that were 9.3% and 6.5%, respectively. Furthermore, the resistance rates of *C. albicans* and *C. glabrata* against itraconazole were 4.8% and 3.6%, respectively. In contrast, Wang et al. in China found that the resistance rates of *C. albicans* against fluconazole and itraconazole were 16.6% and 51.5%, respectively. They also found that the resistance rates of *C. glabrata* to fluconazole and itraconazole were 73.3% and 61.9%, respectively [ 8 ].

In a study carried out by Wang et al., nystatin demonstrated the highest impact against *Candida* spp. However, in the present study nystatin was not available to be examined. The increase in resistance against fluconazole and itraconazole in the aforementioned study can be attributed to the frequent empiric prescription for sporadic VVC, which may result in resistant *C. albicans* and *C. glabrata* causing recurrent VVC infection to emerge. 

Furthermore, it should be mentioned that *C. glabrata* has an inherent resistance to fluconazole too. However, an increase in resistance against antifungal agents may have major consequences resulting in poor outcomes and more chronic forms among women with VVC. Similar results were confirmed by Yassin et al., who reported that *C. glabrata* showed resistance to most used antifungals (i.e., fluconazole, itraconazole, clotrimazole, nystatin, and terbinafine) [ 15 ]. It should be noted that the pattern of antifungal susceptibility in members of the genus *Candida* is different, with the wide use of antifungals in clinical practice. 

One of the limitations of this study was the lack of sufficient same antifungal powders to perform the microdilution broth method simultaneously, and to check the accuracy of the obtained results. Moreover, we did not have any *Candida* strain as quality control and carried out the procedures according to the instructions of the manufacturer of disks.

Hence, the development of high-level and multidrug resistance among *Candida* spp., especially *C. glabrata* has become a global public health concern. Therefore, it is important to know the spp. distribution of *Candida* prior to treatment.

## Conclusion

The results indicated that among the studied *Candida* spp., *C. glabrata*, and *C. albicans* showed the highest resistance to antifungal drugs. Furthermore, the *Candida* spp. isolated from VVC showed the highest rate of sensitivity to voriconazole and ketoconazole and the lowest rate of sensitivity to fluconazole. Further studies on greater sample sizes and more isolates and spp. of *Candida* are recommended.

## Acknowledgments

The authors would like to appreciate the staff of Medical Mycology and Parasitology Laboratory in Gha’em Hospital, Mashhad, Iran. This paper was extracted from a student thesis submitted in partial fulfillment of the requirement for the Master’s degree. It was approved by the Research Committee of the Faculty of Medicine and financially supported by the Research Deputy (No. 931194) of Mashhad University of Medical Sciences, Mashhad, Iran.

## Authors’ contribution

H. Z. interpreted the data and prepared the manuscript. Z. K. collected specimens and performed the project. A. F. designed and planned the study. All authors read and approved the final manuscript.

## Conflicts of interest 

The authors declare that there is no conflict of interest in this study.

## Financial disclosure

The authors declare no financial interests related to the materials of the study.
